# Training to be a spinal endoscopic surgeon: What matters?

**DOI:** 10.3389/fsurg.2023.1116376

**Published:** 2023-03-06

**Authors:** Yizhou Xie, Qun Zhou, Yongtao Wang, Chengzhi Feng, Xiaohong Fan, Yang Yu

**Affiliations:** ^1^ Hospital of Chengdu University of Traditional Chinese Medicine, Chengdu, China; ^2^ School of Nursing, Chengdu University of Traditional Chinese Medicine, Chengdu, China

**Keywords:** spine endoscopic surgery, minimally invasive spine surgery, education, spinal endoscopic surgeon of younger generations, online survey

## Abstract

**Objective:**

Spinal endoscopic surgery has been promoted rapidly in the past decade, attracting an increasing number of young, dedicated surgeons. However, it has long been denounced for its long learning curve as a factor impeding the development of this state-of-the-art technique. The aim of the present study was to discover what really matters in the educational process of becoming a spinal endoscopic surgeon.

**Methods:**

An online survey consisting of 14 compulsory questions was distributed in April and May 2022 through the First Chinese Spinal Endoscopic Surgeons Skills Competition. Reminders were sent to increase response rates.

**Results:**

Of the 893 emails that were sent, we received 637 responses. A total of 375 (76.7%) surgeons most frequently used endoscopic techniques in their practices. Regardless of their different backgrounds, 284 (75.7%) surgeons thought it would be necessary for a young spinal endoscopic surgeon to perform 300 cases independently in order to become proficient, followed by 500 (*n*=43, 11.5%), 100 (*n*=40, 10.7%), and 1,000 (*n*=8, 2.1%) cases. According to the surgeons, the most difficult aspect of mastering the endoscopic technique is a disparate surgical view (*n*=255, 68%), followed by adaption to new instruments (*n*=86, 22.9%) and hand-eye coordination (*n*=34, 9.1%). The most helpful training method for helping the spinal endoscopic surgeons of younger generations improve is operating on simulation models or cadaver courses (*n*=216, 57.6%), followed by online or offline theoretical courses (*n*=67, 17.9%), acquiring opportunities during surgeries (*n*=51, 13.6%), and frequently participating in surgeries as an assistant (*n*=41, 10.9%).

**Conclusion:**

From the perspective of surgeons, to be skilled in spinal endoscopic surgery means overcoming a steep learning curve. However, training systems should be given more attention to make them more accessible to younger surgeons so they can work on simulation models or take cadaver courses.

## Background

Spinal endoscopic surgery, with its unique advantages of a clearer surgical view and continuous irrigation, has been promoted worldwide to treat various forms of spinal disease, from initial degenerative pathology to spinal trauma, infection, and even deformity (1–3). Technically, in terms of the novel instruments being invented steadily with great efforts coming from peers in this field, its surgical approaches and methods have flourished in the past decade to meet the challenges of these diseases (4–6). It is for this reason that spinal endoscopic surgery has attracted an increasing number of young, dedicated surgeons (7).

However, when it comes to this state-of-the-art technique, it is worth mentioning that its learning curve has been long considered a barrier keeping many young surgeons from mastering it (8, 9). Several studies have clarified the steep learning curve and pointed out that it was likely much steeper than any other minimally invasive spinal surgery (10–12). Yet, very few articles have disclosed the reason for this curve and what training techniques young surgeons most urgently need. The aim of the present study was to conduct an online survey in order to analyze the most difficult aspect of learning spinal endoscopic surgery and identify the most beneficial training method for the younger generation of spinal surgeons.

## Methods

A questionnaire was developed using the Tencent Questionnaire Platform for spinal surgeons about the relevant educational issues of spinal endoscopic surgery (Supplementary Material 1). The study samples were targeted at the members of the First Chinese Spinal Endoscopic Surgeons Skills Competition, which is a national academic competition for spinal endoscopic surgery. Spinal endoscopic surgery has a predominant status in the field of minimally invasive spinal surgery in China, and this nationwide competition was held to represent surgeons and allied health professionals dedicated to advancement in this field (13, 14). As the corresponding author’s institution was also the participating and reviewing institution, 2,893 members were contacted *via* email. Questionnaires were sent out in April and May 2022, with reminders after 2 weeks to increase the response rate.

### Screening for surgeons

To make the study more representative, the participants involved in this survey were the surgeons who frequently utilized spinal endoscopy in their daily practice. Hence, surgeons who did not specialize in spinal surgery or who did not regularly use spinal endoscopy in their daily practice were excluded from the study.

### Survey content

The survey consisted of 14 compulsory questions for surgeons. The first six questions were about basic personal information such as background, age, gender, training specialty, and title. Questions 7–11 were to acknowledge the status of their application on spinal endoscopic surgery. The last three questions collected the surgeons’ attitudes toward endoscopic education.

### Statistical analyses

For statistical analyses, IBM SPSS version 25.0 (IBM Corp., Armonk, NY, USA) was used. The age of the participants was represented as the mean and standard deviation ($\bar{x}\pm s$) while other indexes were expressed as a percentage [*n* (%)].

## Results

**Figure 1:**
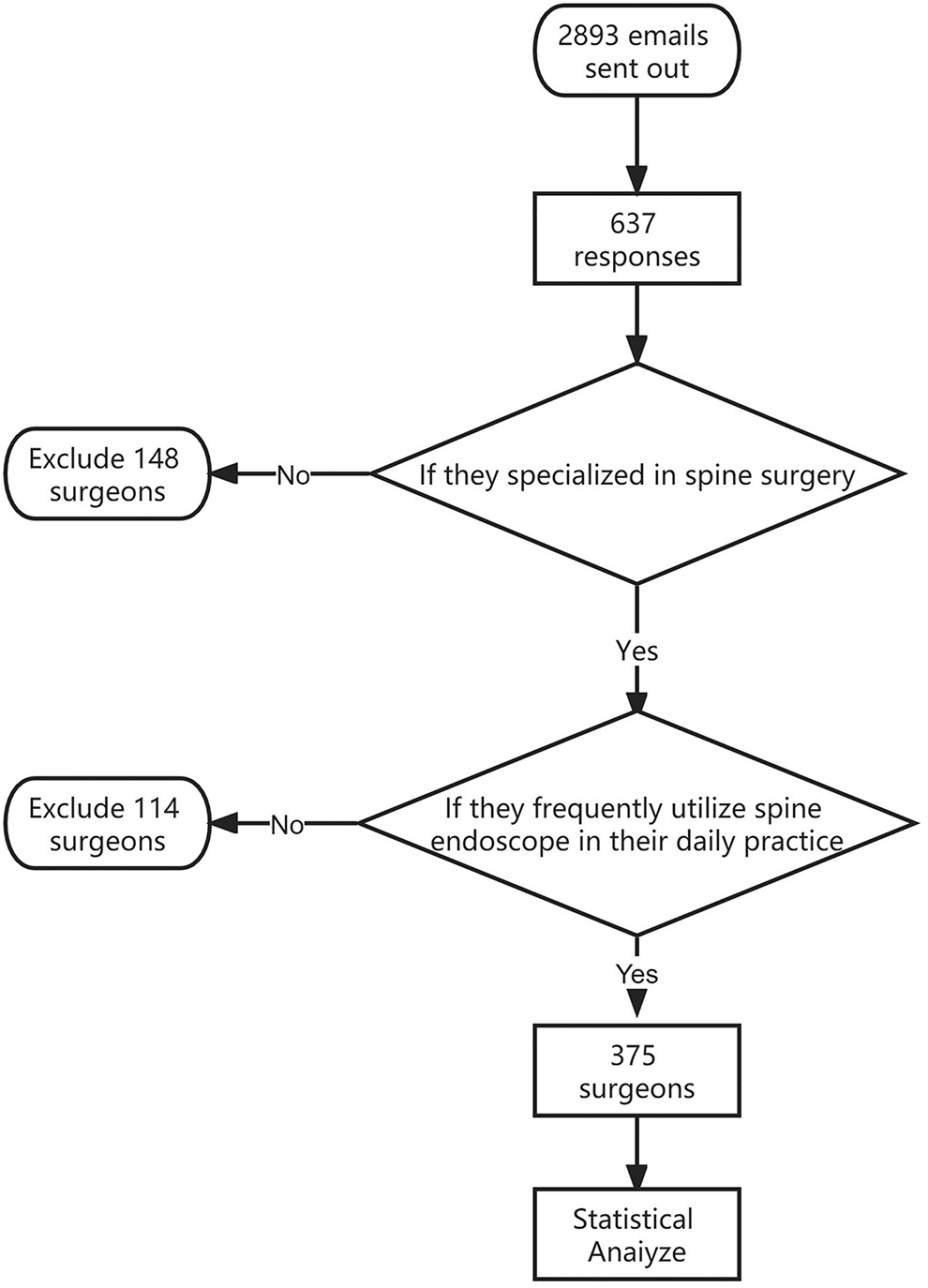
Screening process for surgeons.

**Figure 2:**
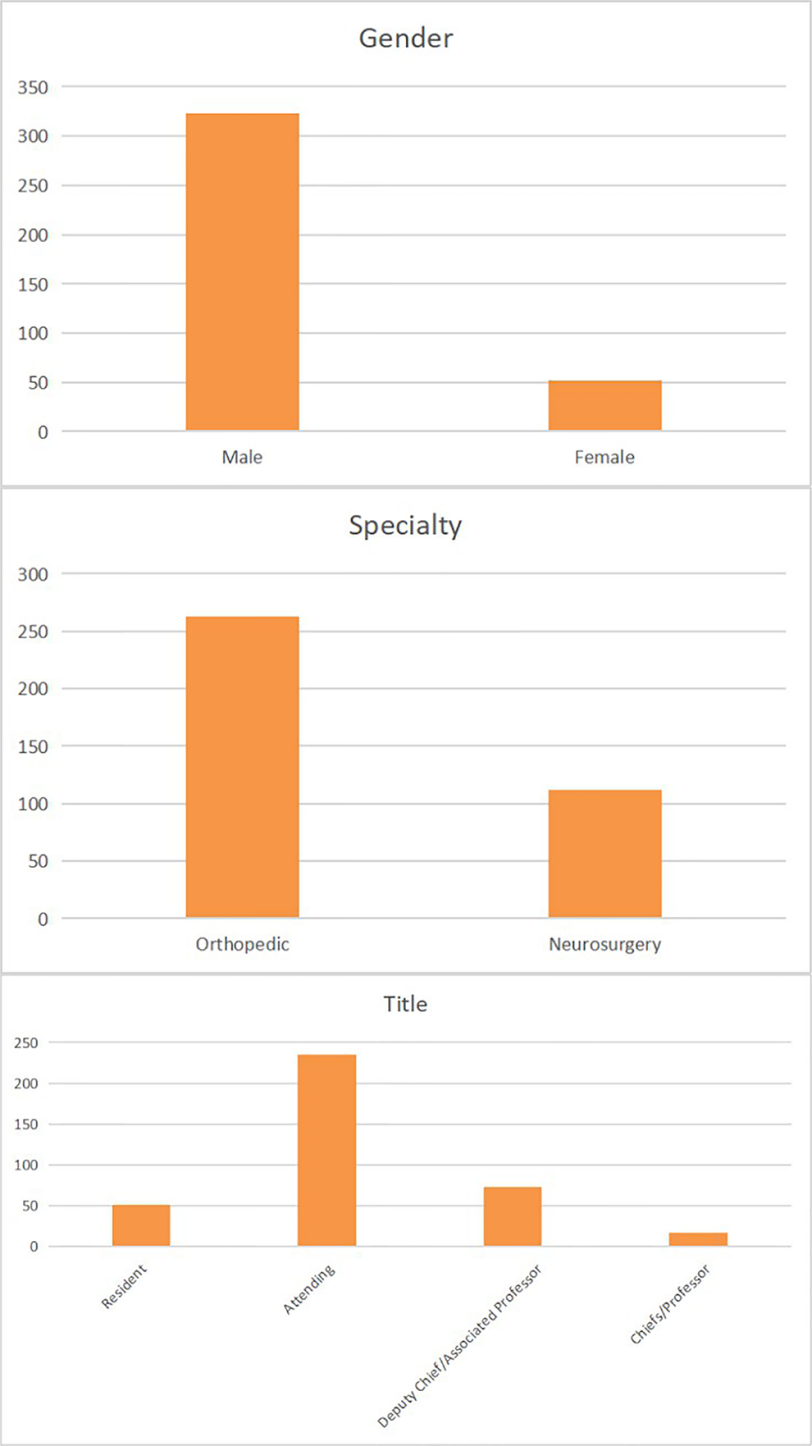
The basic background information of 375 surgeons.

Of the 893 emails that were sent, we received 637 responses. All respondents specialized in spinal surgery; with 489 (76.8%) considering minimally invasive spinal surgery to be their subspecialty. Among them, 375 (76.7%) surgeons used the endoscopic technique most frequently in their practice. The screening process for surgeons is shown in Figure 1. Of these 375 surgeons (mean age 42.5 ± 4.3 years) 323 (86.1%) were men and 52 (13.9%) were women. Of them, 263 (70.1%) were from an orthopedic background while 112 (29.9%) were from a neurosurgery background. In terms of titles, 51 (13.6%) were residents, followed by 235 (62.7%) attendings, 73 (19.5%) deputy chiefs/associated professors, and 16 (4.3%) chiefs/professors (Figure 2).

**Figure 3:**
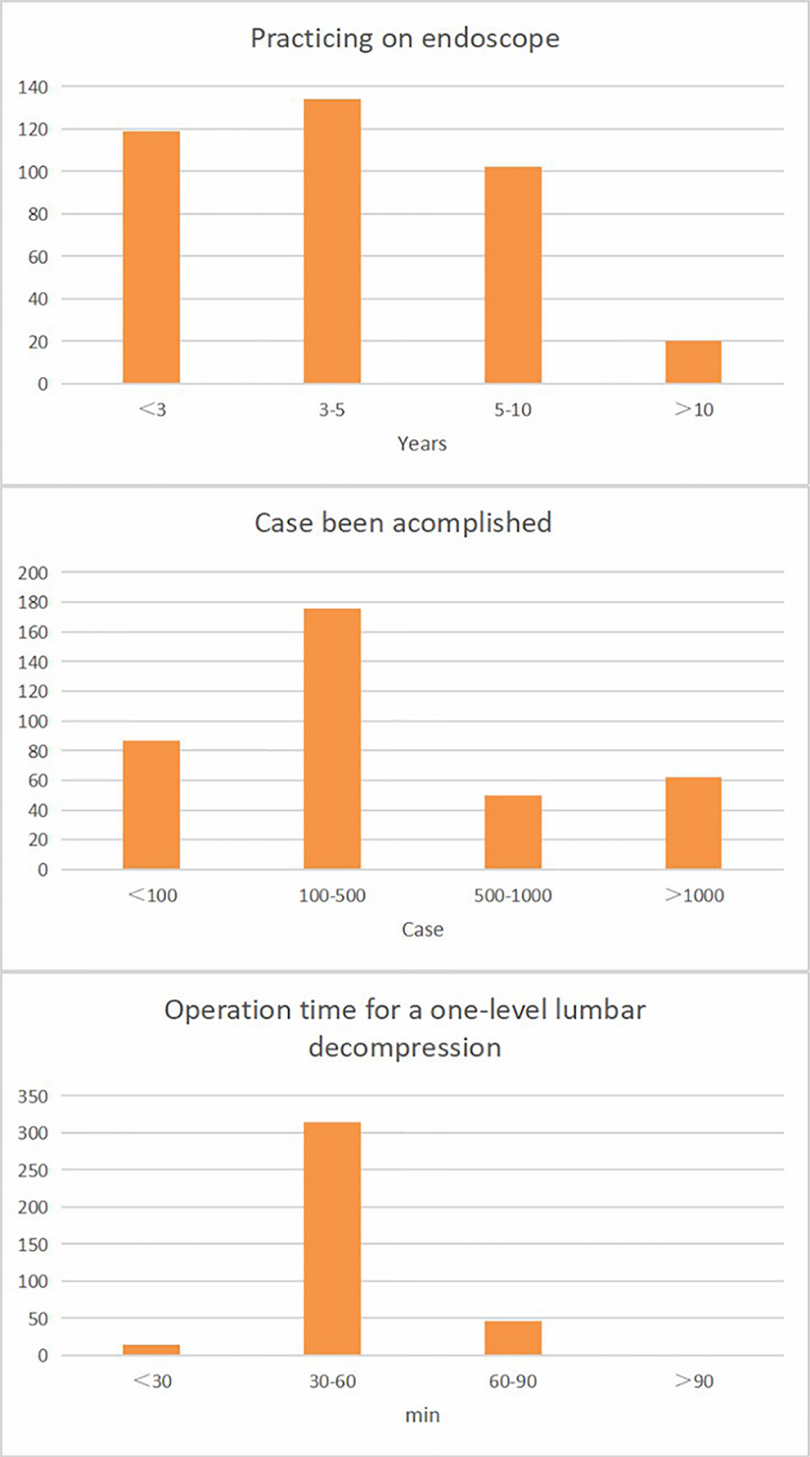
Spine endoscopic application status of 375 surgeons.

With regard to their years of experience with the endoscopic application, 119 (31.7%) surgeons had been practicing endoscopic surgery for less than 3 years, 134 (35.7%) had been practicing for 3–5 years, 102 (27.2%) had been practicing for 5–10 years, and only 20 (5.3%) surgeons had been practicing for more than 10 years. Concerning the number of cases they had already completed, 87 (23.2%) of the surgeons had worked on less than 100 cases, 176 (46.9%) had already done 100–500 cases, 50 (13.3%) had already done 500–1,000 cases, and 62 (16.5%) of them had already done more than 1,000 cases. In terms of the operating time for a one-level lumbar decompression, 15 (4%) of the surgeons could complete the procedure in less than 30 minutes, 314 (83.7%) in 30–60 minutes, 46 (12.3%) in 60–90 minutes, and none of them took more than 90 minutes (Figure 3).

**Figure 4:**
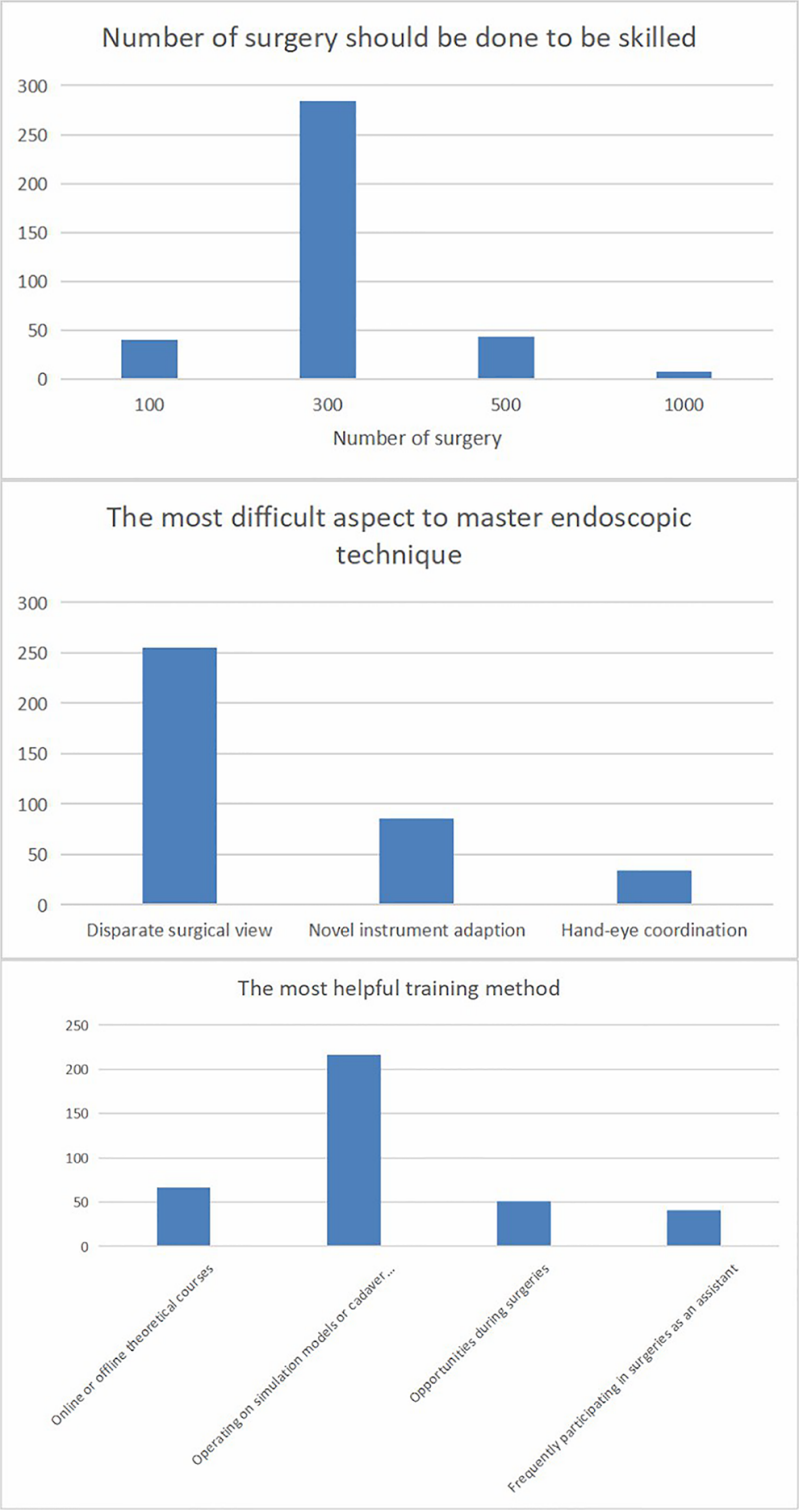
Attitude towards education process from 375 surgeons.

Regarding the surgeons’ attitudes toward the education process for spinal endoscopic surgeons, 284 (75.7%) of them thought it would be necessary for a young spinal endoscopic surgeon to perform 300 cases independently to become proficient, followed by 500 (*n*=43, 11.5%) cases, 100 (*n*=40, 10.7%) cases, and 1,000 (*n*=8, 2.1%) cases. According to the surgeons, the most difficult aspect of this technique to master was the disparate surgical view (*n*=255, 68%), followed by adapting to the novel instruments (*n*=86, 22.9%) and hand-eye coordination (*n*=34, 9.1%). The most helpful training method to help the spinal endoscopic surgeon of the younger generation improve was operating on simulation models or cadaver courses (*n*=216, 57.6%), followed by online or offline theoretical courses (*n*=67, 17.9%), acquiring opportunities during surgeries (*n*=51, 13.6%), and frequently participating in surgeries as an assistant (*n*=41, 10.9%) (Figure 4).

## Discussion

The steep learning curve of this state-of-the-art technique has long been mentioned in many previous studies (10–12). Kotheeranurak et al. conducted an online survey about the learning curve, motivation, and obstacles of full-endoscopic spinal surgery in Thailand. They drew the conclusion that the trend of endoscopic spinal surgery has continued to grow and that the appropriate number of cases until one felt confident was approximately 28. The primary motivator and obstacles were personal interest and lack of support (15). Hsu et al. retrospectively evaluated the clinical presentation of 57 patients who underwent full-endoscopic lumbar discectomy and 66 patients who underwent open microdiscectomy using Spearman's coefficient of rank correlation (rho) to assess the learning curves for the transforaminal and interlaminar procedures of full-endoscopic lumbar discectomy. They believed that the transforaminal approach had a steep and easy learning curve, whereas the learning curve of the interlaminar approach was deemed flat and difficult (16). Gadjradj et al. conducted a study by observing the clinical outcomes during and after the learning curve (20 cases) presented by three surgeons new to spinal endoscopy. They later determined that spinal endoscopic surgery had a relevant steep learning curve and that young spinal surgeons should use the endoscope under the supervision of a senior surgeon (17).

In order to uncover the deeper factor for this challenging issue, the present study conducted a survey of 375 spinal endoscopic surgeons to reveal what matters to the growth of a young, medical professionals. Through the screening based on questions 3–7, it was determined that they were all peers who were dedicated specifically to this method. It was critical that the survey be representative. Despite their varied backgrounds, most of the respondents performed well in endoscopic surgery according to questions 8–10 of the survey. The study pointed out that the majority of surgeons thought the threshold for a surgeon to be skilled in spinal endoscopy was at least 300 cases. In fact, surgeons may face many challenges in the initial phase of practicing this technique, such as a lengthy operating time, a high complication rate, and even failure to finish the procedure (18–20). However, they should be patient and persevere rather than be dejected in order to add to their experiences regularly. There is also evidence that the situation will improve over time (17). According to the surgeons, the most difficult aspect of mastering this technique is the disparate surgical view (21). Unlike conventional open surgery or the cadaver specimens that medical students see regularly in medical school, the surgical view from a spinal endoscopy is entirely different because its limited view with the amplified image by the lens will make the normal-sized anatomic sites appear fairly magnified, which might confuse the beginners when recognizing the anatomic landmarks (22). Hence, great importance should be given to training. Based on the survey, the most beneficial training method for surgeons was operating on simulation models or cadaver courses. This conveyed a vital message to organizations and manufacturers who are dedicated to promoting the technique and helping less-experienced surgeons. Even though cadavers are not always available, especially in some regions, it is satisfying to see peers develop innovative alternative training programs (23, 24). This trend is in line with the survey, and it should be maintained for our efforts to be worthwhile.

## Conclusion

From the perspective of surgeons, to be skilled in spinal endoscopic surgery means overcoming a steep learning curve. However, more importance should be attached to training systems so that younger generations of surgeons can operate on simulation models or take cadaver courses.

## Data Availability

The raw data supporting the conclusions of this article will be made available by the authors, without undue reservation.
