# Supplementary material for: Training to be a spinal endoscopic surgeon: What matters?
Source: Front Surg. 2023 Mar 6;10:1116376. doi: 10.3389/fsurg.2023.1116376 (PMC10025468; doi:10.3389/fsurg.2023.1116376)
Supplement: Supplementary file 1 [file Datasheet1.pdf]

Online Survey for Situation of Spine Endoscopic Education

If you have any questions about the questionnaire, please do not hesitate to contact us.

1.**What is your age? ** *

2.**What is your gender?** *

◯ Male

◯ Female

3.**Were you trained from orthopedic side or neurosurgery side ?** *

◯ Orthopedic

◯ Neurosurgery

4.**What is your title?** *

◯ Resident

◯ Attending

◯ Deputy Chief/Associated Professor

◯ Chief/Professor

5.**Are you specialized in spine surgery now?** *

◯ Yes

◯ No

6.**Is Minimally Invasive Spine Surgery (MISS) your subspecialty?** *

Yes

No

7.**What sort of MISS technique you most frequently use in your practice?** *

Endoscope

Microscope

MED

8.**How long have you been practicing on endoscopic surgery?** *

< 3y

3-5y

5-10y

> 10y

9.**How many cases have you already been done for endoscopic surgery?** *

< 100

100-500

500-1000

> 1000

10.**How long does it usually take for you to finish a one-level lumbar decompression? (min)** *

< 30

30-60

60-90

> 90

11.**What the number of surgeries do you think is necessary for a spine endoscopic surgeon to become proficient?** *

- ◯ 50
- ◯ 100
- ◯ 300
- ◯ 500
- ◯ 1000

12.**What aspect do you regard as the most difficult part to master endoscopic technique?** *

- ◯ Hand-eye coordination
- ◯ Novel instruments adaption
- ◯ Disparate surgical view

13.**What sort of training method do you think is the most helpful to facilitate spine endoscopic surgeon of younger generation to improve?** *

- ◯ Online or offline theoretical courses
- ◯ Frequently participating in surgeries as an assistant
- ◯ Acquiring the opportunities during surgeries
- ◯ Operating on simulation models or cadaver courses

Copyright © 1998-2022 Tencent.
